# Reporting of participant flow diagrams in published reports of randomized trials

**DOI:** 10.1186/1745-6215-12-253

**Published:** 2011-12-05

**Authors:** Sally Hopewell, Allison Hirst, Gary S Collins, Sue Mallett, Ly-Mee Yu, Douglas G Altman

**Affiliations:** 1 Centre for Statistics in Medicine, University of Oxford, Wolfson College, Linton Road, Oxford, UK

## Abstract

**Background:**

Reporting of the flow of participants through each stage of a randomized trial is essential to assess the generalisability and validity of its results. We assessed the type and completeness of information reported in CONSORT (Consolidated Standards of Reporting Trials) flow diagrams published in current reports of randomized trials.

**Methods:**

A cross sectional review of all primary reports of randomized trials which included a CONSORT flow diagram indexed in PubMed core clinical journals (2009). We assessed the proportion of parallel group trial publications reporting specific items recommended by CONSORT for inclusion in a flow diagram.

**Results:**

Of 469 primary reports of randomized trials, 263 (56%) included a CONSORT flow diagram of which 89% (237/263) were published in a CONSORT endorsing journal. Reports published in CONSORT endorsing journals were more likely to include a flow diagram (62%; 237/380 versus 29%; 26/89). Ninety percent (236/263) of reports which included a flow diagram had a parallel group design, of which 49% (116/236) evaluated drug interventions, 58% (137/236) were multicentre, and 79% (187/236) compared two study groups, with a median sample size of 213 participants. Eighty-one percent (191/236) reported the overall number of participants assessed for eligibility, 71% (168/236) the number excluded prior to randomization and 98% (231/236) the overall number randomized. Reasons for exclusion prior to randomization were more poorly reported. Ninety-four percent (223/236) reported the number of participants allocated to each arm of the trial. However, only 40% (95/236) reported the number who actually received the allocated intervention, 67% (158/236) the number lost to follow up in each arm of the trial, 61% (145/236) whether participants discontinued the intervention during the trial and 54% (128/236) the number included in the main analysis.

**Conclusions:**

Over half of published reports of randomized trials included a diagram showing the flow of participants through the trial. However, information was often missing from published flow diagrams, even in articles published in CONSORT endorsing journals. If important information is not reported it can be difficult and sometimes impossible to know if the conclusions of that trial are justified by the data presented.

## Background

The CONSORT (CONsolidated Standards Of Reporting Trials) Statement, most recently updated in 2010, is an evidence-based, minimum set of recommendations for reporting the findings of randomized trials [1,2] It offers a standard way for authors to prepare reports of trial findings, facilitating their complete and transparent reporting, and aiding their critical appraisal and interpretation. The statement consists of a checklist of items to be addressed in the text of a report of a randomized trial, especially the methods and results sections, and a flow diagram which shows the flow of participants through each stage of a trial. There is evidence to show that journal adoption of the CONSORT Statement leads to improvements in the reporting of randomized trials [3], aiding their critical assessment by readers.

For some randomized trials the flow of participants through each phase of the trial can be relatively straightforward to describe, particularly where there were no losses to follow-up or exclusions. However, in more complex trials it may be difficult for readers to discern whether and why some participants did not receive the treatment as allocated, were lost to follow-up, or were excluded from the analysis [4]. This information is crucial as participants who were excluded after allocation are unlikely to be representative of all participants in the study [5]. A completed flow diagram is especially valuable for such trials.

**Figure 1:**
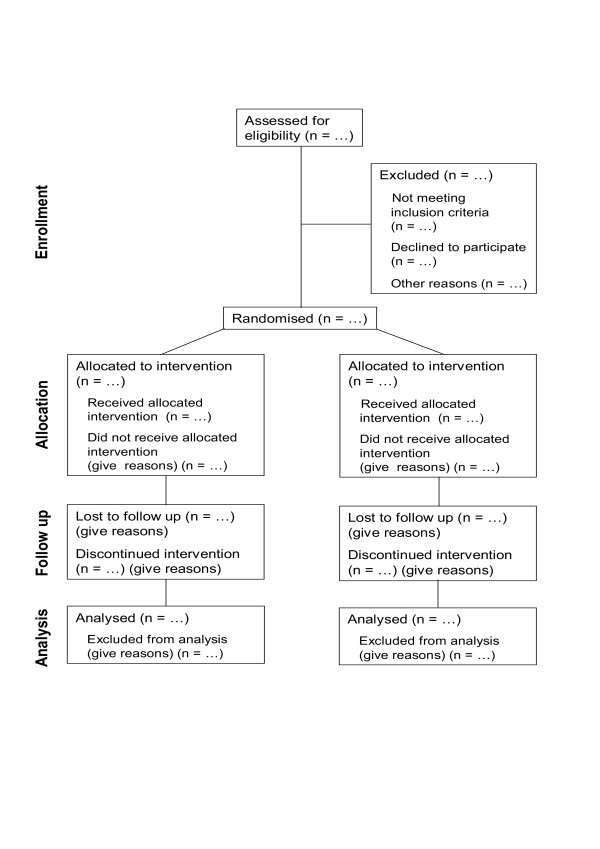
**Flow diagram of the progress through the phases of a 2-group parallel randomized trial (1;2)**.

The template for the CONSORT flow diagram [1,2] is shown in Figure 1. Information required to complete a CONSORT flow diagram includes the number of participants evaluated for potential enrolment into the trial (if known) and the number excluded at this stage either because they did not meet the inclusion criteria or declined to participate. It also requires for each intervention group the numbers of participants who were randomly assigned, received treatment as allocated, completed treatment as allocated, and were included in the main analysis, with numbers and reasons for exclusions at each step.

If important information is not reported it is often not possible to tell which participants received which intervention and how they were included in the analysis, making it difficult or sometimes impossible to know whether the conclusions of the trial are justified by the data [6]. A complete CONSORT flow diagram reduces the time for readers to find the essential information to assess the reliability of a trial. It is also likely to improve the availability of some information which otherwise might not be reported.

The aim of our study was to provide a comprehensive assessment of the type and completeness of information reported in CONSORT flow diagrams published in current reports of randomized trials. We also examined a random sample of reports, for which some items were missing from the CONSORT flow diagram, to see whether appropriate information was reported elsewhere in the text and how much time was required to identify this additional information. We did not examine the text of reports that did not include a flow diagram.

## Methods

### Sample

We searched PubMed for all reports of randomized trials indexed from 1 July to 31 December 2009 with the publication type "Randomized Controlled Trial" (search as of 4 January 2010). This search strategy was chosen based on a review of published search filters for retrieving reports of randomized trials from MEDLINE, which found that the publication type term "Randomized Controlled Trial" performed well with 93% sensitivity, 98% specificity and 56% precision [7]. We limited our search to the National Library of Medicine's set of core clinical journals (Abridged Index Medicus) as these journals are typical of those accessed by busy clinicians. All journals published in this set are in English.

### Eligibility criteria

We included all primary reports of a randomized trial defined as a prospective study assessing health-care interventions in human participants who were randomly allocated to study groups. We included all studies of parallel group, crossover, cluster, factorial and split-body design. If a publication described more than one report of a randomized trial, we included the first trial or the trial including the most information. Interim analysis, secondary publications, editorials, letters, studies of cost-effectiveness and diagnostic test accuracy reports were excluded.

### Review progress

One person (SH) screened the titles and abstracts of all retrieved reports to exclude any obvious ineligible studies (i.e. not trials). A copy of the full report was then assessed for all remaining articles. Any additional material about the trial included as an appendix on the journal website was also obtained, if available. All reports were then classified as either including a CONSORT flow diagram [1,2] (eligible) or not including a CONSORT flow diagram (ineligible).

### Data extraction

Data extraction was carried out by five reviewers working in pairs (in blocks of 50 articles allocated at random). Each pair independently extracted data from eligible reports; any differences between reviewers were resolved by discussion with the involvement of an arbitrator if necessary. To ensure consistency between reviewers, they all piloted the data extraction form using a sample of 10 papers from the sample under review. A data extraction manual was developed to provide guidance for each item on the data extraction form.

For each eligible report we extracted information on the trial design, journal type, medical specialty, type of intervention, number of data collection sites, number of randomized groups, sample size and whether the report was published in a CONSORT endorsing journal based on the journals' 'Instructions to Authors' (assessed November 2010).

We extracted from the flow diagram whether information (either directly reported or could be inferred from the flow diagram) was provided on the number of participants:

• assessed for eligibility (overall), excluded as they did not meet the inclusion criteria, excluded as they met inclusion criteria but declined to participate, or excluded for other reasons;

• randomized (overall);

• allocated to intervention (per group), received the allocated intervention (per group), did not receive the allocated intervention (per group), and reasons they did not receive the allocated intervention (per group);

• lost to follow-up (per group), reasons for lost to follow up (per group), discontinued intervention (per group), and reasons for discontinued intervention (per group);

• included in main analysis (per group), excluded from analysis (per group), and reasons excluded from analysis (per group);

We also extracted data on any additional information included in the flow diagram (overall or per group).

For incomplete participant flow diagrams (i.e. those which had items missing or flagged as unclear) we examined a random sample (25%; n = 54) of reports to see whether the missing information was reported elsewhere in the text instead. We also assessed how long it took to identify this additional information, if reported.

### Data analysis

The primary analysis was focused on parallel group trials; two group and multi-arm. We calculated the proportion (%) of parallel group trial publications reporting specific items included in the CONSORT flow diagram. We also carried out an analysis on the reporting of cluster trials which included a participant flow diagram based on the guidance in the CONSORT extension for cluster trials [8].

## Results

**Figure 2:**
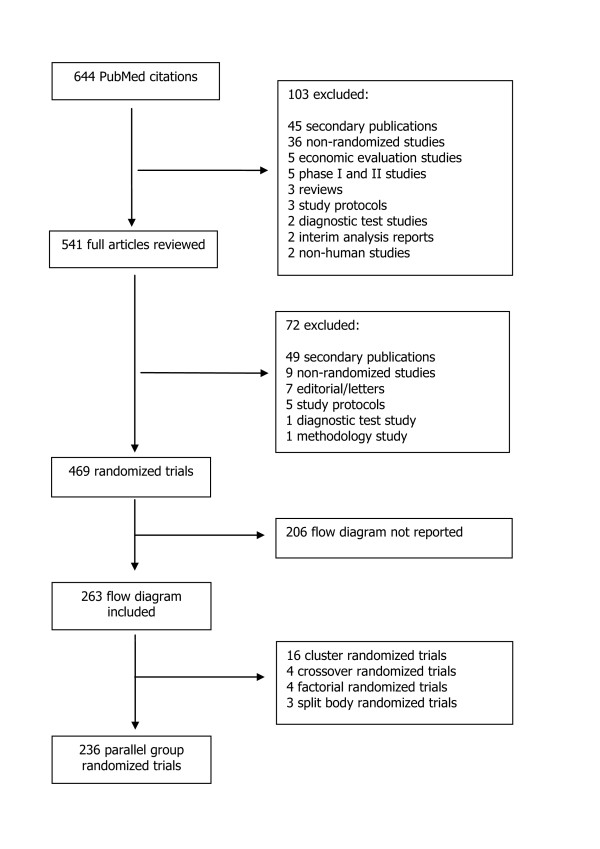
**Identification of randomized trials from PubMed citations indexed from July to December 2009**.

The PubMed publication type search term "Randomized Controlled Trial" identified 644 possible reports of randomized trials. After screening the titles and abstracts of all retrieved citations, we reviewed 541 full text articles resulting in 469 primary reports of randomized trials (Figure 2); of these 380 were published in journals which referred to the CONSORT Statement in their journal's Instruction to Authors and 89 were published in journals which did not refer to CONSORT. Sixty-two percent (237/380) of reports published in a CONSORT endorsing journal included a CONSORT flow diagram compared to only 29% (26/89) of reports published in a non CONSORT endorsing journal.

Thus, 263 (56%) of the 469 primary reports of randomized trials included a CONSORT flow diagram; 236/263 (90%) were reports of parallel group trials, 16 (6%) were cluster randomized, four (1.5%) were crossover, four (1.5%) were factorial and three (1%) were split body trials (Figure 2). The primary trial reports were published in 50 different journals, with the majority appearing in specialty journals. The journals with the most reports in the sample were the Lancet (n = 27), New England Journal of Medicine (n = 23), BMJ (n = 20), JAMA (n = 18) and Pediatrics (n = 16). Ninety percent (237/263) of articles included in our cohort were published in journals which referred to the CONSORT Statement in their journal's Instruction to Authors; 41/50 (82%) journals. In comparison 69% (143/206) of articles which did not include a flow diagram, thus excluded from our analysis, were published in journals which referred CONSORT Statement in their journal's Instruction to Authors; 25/55 (45%) journals.

Table 1 provides information on reporting of general trial characteristics. Around half (49%; n = 116) of parallel group trials investigated drugs as the primary intervention of interest, 20% (n = 46) assessed surgical or procedural interventions, 27% (n = 64) assessed counselling or lifestyle interventions and the remaining 4% (n = 10) assessed equipment/devices. Thirty-one percent (n = 72) of parallel group trial reports explicitly stated that they were single centre trials and 58% (n = 137) stated that they were multicentre; the number of study centres was not explicitly defined in the remaining reports (n = 27). The majority of parallel group trials (79%; n = 187) had two study arms. The median number of participants recruited per parallel group trial was 213 (10^th ^to 90^th ^percentile 50 to 1217); this was smaller than for the group "other" trials (median 529; 10^th ^to 90^th ^percentile 56 to 61280) due to the inclusion of cluster randomized trials.

**Table 1:** General characteristics of randomized trials reporting a flow diagram indexed in PubMed from July to December 2009 (n = 263)

Trial design	Parallel (n = 236)	Other * (n = 27)
**Journal type**		
Specialty	144 (61%)	11 (41%)
General medical	92 (39%)	16 (59%
**Funding**		
Solely industry	40 (17%)	1 (4%)
Part industry	43 (18%)	2 (7%)
Non industry	126 (53%)	20 (74%)
None	5 (2%)	2 (7%)
Unknown	22 (9%)	2 (7%)
**CONSORT endorsing journal**		
Yes	210 (89%)	27 (100%)
No	26 (11%)	0 (0%)
**Top 5 common specialty fields**		
	Paediatrics 34 (14%)	Paediatrics 10 (34%)
	Cardiology 29 (12%)	Cardiology 2 (7%)
	Psychiatry 20 (9%)	Psychiatry 2 (7%)
	Infectious diseases 20 (9%)	Infectious diseases 2 (7%)
	Oncology 17 (7%)	Oncology 1 (3%)
**Intervention**		
Drug	116 (49%)	6 (22%)
Surgery/procedure	46 (20%)	3 (11%)
Counselling/lifestyle	64 (27%)	15 (56%)
Equipment	10 (4%)	3 (11%)
**Study centres**		
Single	72 (31%)	4 (15%)
Multiple	137 (58%)	20 (74%)
Unclear	27 (11%)	3 (11%)
**Number of study groups**		
2	187 (79%)	20 (74%)
3	36 (15%)	2 (7%)
4	11 (5%)	3 (11%)
> 4	2 (1%)	2 (7%)
**Sample size**		
Median (IQR)	213 (101 to 476)	529 (130 to 2243) †
10 to 90 percentile	50 to 1217	56 to 61280

* Cluster n = 16; Crossover n = 4; Factorial n = 4; Split Body n = 3

† Sample size not reported for 1 cluster trial

Sources of funding were provided in most trial reports; 17% (n = 40) of parallel group trials were funded solely by industry, 18% (n = 43) were part industry funded and 53% (n = 126) were non industry funded. For comparison, information on reporting of general trial characteristics for non parallel group trials is also provided in Table 1.

### Information included in CONSORT flow diagrams for parallel group trials

Table 2 provides a summary of the information reported (either directly reported or could be inferred from the flow diagram) in the CONSORT flow diagram for the 236 reports of parallel group trials which included a participant flow diagram. Eighty-one-percent (n = 191) of flow diagrams reported the overall number of people assessed for eligibility, 71% (n = 168) reported the overall number of people excluded prior to randomization and 98% (n = 231) reported the overall number randomized. Reasons for exclusion prior to randomization were less well reported; only 57% (n = 136) reported the number of people excluded because they did not meet the inclusion criteria for the trial, 66% (n = 157) reported the number excluded because they declined to participate in the trial and 60% (n = 141) reported the number excluded for other reasons.

**Table 2:** Reporting of CONSORT flow diagram items for parallel group trials (n = 236)

	Reported *	Not reported	Unclear
**Enrolment**			
Assessed for eligibility (overall)	191 (81%)	43 (18%)	2 (1%)
Excluded (overall)	168 (71%)	64 (27%)	4 (2%)
Not meeting inclusion criteria	136 (57%)	54 (23%)	46 (20%)
Declined to participate	157 (66%)	56 (24%)	23 (10%)
Other reasons	141 (60%)	55 (23%)	40 (17%)
Randomized (overall)	231 (98%)	2 (1%)	3 (1%)
**Allocation**			
Allocated to intervention (per group)	223 (94%)	7 (3%)	6 (3%)
Received allocated intervention (per group)	95 (40%)	120 (51%)	21 (9%)
Did not receive allocated intervention (per group)	91 (38%)	117 (50%)	28 (12%)
Reported reasons did not receive intervention	76 (32%)	133 (55%)	27 (11%)
**Follow up**			
Lost to follow up (per group)	158 (67%)	47 (20%)	31 (13%)
Reported reasons lost to follow up	93 (40%)	116 (49%)	27 (11%)
Discontinued intervention (per group)	145 (61%)	46 (20%)	45 (19%)
Reported reasons discontinued intervention	128 (54%)	65 (28%)	42 (18%)
**Analysis**			
Included in main analysis (per group)	128 (54%)	108 (46%)	
Excluded from main analysis (per group)	82 (35%)	154 (65%)	
Reported reasons excluded	78 (33%)	128 (67%)	

* Directly reported or could be inferred from the flow diagram.

The majority (94%; n = 223) of flow diagrams reported the number of participants allocated to each arm of the trial, however less than half (40%; n = 95) reported the number of participants in each arm of the trial who actually received the allocated intervention. Just over a third (38%; n = 91) of flow diagrams reported the number of participants who did not receive the allocated intervention; only 32% (n = 76) gave the reason for exclusion, for example, withdrawal of patient consent or change in initial patient diagnosis. Sixty-seven percent (n = 158) of flow diagrams included the numbers lost to follow up in each arm of the trial; under half (40%; n = 93) reported the reason for loss to follow up for example the patient had moved and could not be located. Similarly, 61% (n = 145) of flow diagrams reported how many participants discontinued the intervention during the trial; 54% (n = 128) reported the reason for discontinuation for example death of the patient, adverse effects of the intervention or for personal reasons. Just over half (54%; n = 128) of flow diagrams reported the number of participants included in the analysis and few (35%; n = 82) explicitly stated the number excluded from the analysis; or the reason for exclusion from the analysis (33%; n = 78).

In addition to the information currently required to complete a CONSORT flow diagram we also assessed any additional information that was included. Among the 236 flow diagrams, 10 (4%) reported the overall number of participants screened prior to eligibility assessment, 20 (8%) reported the number of participants lost to follow up in each arm of the trial for more than one time point and four (2%) reported the number of participants in each arm of the trial included in both an intention to treat and per protocol analysis. We also noted some instances where the number of participants didn't add up across the flow diagram; however we didn't assess this systematically across all trials. Interestingly, despite approximately half of trial reports assessing non drug interventions (Table 1), no flow diagram reported the number of care providers or centres performing the intervention or the number of patients treated by each care provider or in each centres - as recommended in the CONSORT extension for non-pharmacological interventions [9].

While Table 2 provides a summary of the number and proportion of trials explicitly reporting different CONSORT flow diagrams items, there were many instances where an item was either missing from the flow diagram or where the information reported for that item was unclear. A good example of the latter is that authors sometimes combined reporting of loss to follow and discontinuation of the treatment intervention and did not differentiate between them. Similarly, authors sometimes combined the reasons for exclusion prior to randomization and so the exact reasons for exclusions (e.g. not meeting inclusion criteria, declined to participate) were unclear.

### Information included in CONSORT flow diagrams for cluster randomized trials

Table 3 provides a summary of the information reported in the CONSORT flow diagram for the subset of 16 cluster trials that included a participant flow diagram. Guidance in the CONSORT extension for presenting cluster trials [8] suggests that the information in the diagram might vary depending on the type of analysis. We assessed for each item whether the flow diagram reported the number of clusters, the number (e.g. total, median or mean) of participants or both. There was considerable variability in the type and level of detail reported.

**Table 3:** Reporting of CONSORT flow diagram items for cluster trials (n = 16)

	Reported *	Cluster level	Patient level ≠	Not applicable
**Enrolment**				
Assessed for eligibility (overall)	12 (75%)	8/12	4/12	
Excluded (overall)	11 (69%)	7/11	4/11	1/11
Not meeting inclusion criteria	8 (50%)	2/8	5/8	1/8
Declined to participate	11 (69%)	3/11	2/11	6/11
Other reasons	10 (63%)	4/10	3/10	4/10
Randomized (overall)	15 (94%)	13/15	4/15	
**Allocation**				
Allocated to intervention (per group)	15 (94%)	14/15	8/15	
Received allocated intervention (per group)	14 (88%)	8/14	14/14	
Did not receive allocated intervention (per group)	14 (88%)	6/14	10/14	
Reported reasons did not receive intervention	12 (75%)	4/12	9/12	
**Follow up**				
Lost to follow up (per group)	13 (81%)	6/13	12/13	
Reported reasons lost to follow up	11 (69%)	3/11	9/11	
Discontinued intervention (per group)	10 (63%)	2/10	3/10	6/10
Reported reasons discontinued intervention	10 (63%)	1/10	3/10	6/10
**Analysis**				
Included in main analysis (per group)	15 (94%)	9/15	14/15	
Excluded from main analysis (per group)	12 (75%)	2/12	7/12	4/12
Reported reasons excluded	12 (75%)	1/12	7/12	4/12

* Directly reported or could be inferred from the flow diagram.

≠Patient level: 10 flow diagrams reported the total number of participants, 2 reported the median and range, 1 reported the mean and total number of participants, 1 reported the mean and standard deviation, 1 reported the average and range and 1 reported the median, range and total number of participants. Some flow diagrams reported both the number of clusters per item and the number of participants.

### Reporting of additional information in the text of the full publication in parallel group trials

Only 18 of the 236 flow diagrams had complete information. In the final part of the study we sought to identify whether information not reported in the flow diagram was reported in the text of the full publication and if so, how long it took to locate. Across the 236 reports of parallel group trials the median number of items either not reported or flagged as unclear based on the information in the flow diagram was seven out of a possible 17 items (IQR 4 to 10); 41% (IQR 24% to 59%).

We examined a subset (25%; n = 54/218) of reports where information was either not reported or unclear in the flow diagram to see if it was available elsewhere in the text or in tables or figures. Based only on the information reported in the 54 flow diagrams the median number of items either not reported or unclear was seven (IQR 3 to 11); 41% (IQR 18% to 65%). Information retrieved from the full text reduced the number of items which were either missing or unclearly reported to a median of four items per flow diagram (IQR 1 to 6); 24% (IQR 6% to 35%). Improvement generally focused on the clarification of reasons for exclusion and the numbers of participants included and excluded from the analysis (Table 4). Of particular interest, while only half (50%; n = 27/52) of flow diagrams provided information on the number of participants included in the main analysis this increased to 94% (n = 51/54) when examining the full text and relevant tables and figures. However, it required more time on the part of the reader to extract this information. Overall the median time required to extract additional information from the full article was six minutes (IQR 4 to 10), although there was some variation depending on the expertise of the reviewer.

**Table 4:** Additional information reported in the text of the full publication for parallel group trials (n = 54)

	Reported * in flow diagram only	Reported * in flow diagram or text
**Enrolment**		
Assessed for eligibility (overall)	43 (80%)	44 (81%)
Excluded (overall)	40 (74%)	45 (83%)
Not meeting inclusion criteria	28 (52%)	37 (68%)
Declined to participate	34 (63%)	39 (72%)
Other reasons	29 (54%)	39 (72%)
Randomized (overall)	51 (94%)	52 (96%)
**Allocation**		
Allocated to intervention (per group)	49 (91%)	52 (96%)
Received allocated intervention (per group)	22 (41%)	33 (61%)
Did not receive allocated intervention (per group)	19 (35%)	29 (54%)
Reported reasons did not receive intervention	17 (31%)	28 (52%)
**Follow up**		
Lost to follow up (per group)	37 (68%)	43 (80%)
Reported reasons lost to follow up	22 (41%)	29 (54%)
Discontinued intervention (per group)	34 (63%)	38 (70%)
Reported reasons discontinued intervention	30 (55%)	37 (68%)
**Analysis**		
Included in main analysis (per group)	27 (50%)	51 (94%)
Excluded from main analysis (per group)	20 (37%)	48 (89%)
Reported reasons excluded	18 (33%)	42 (77%)

* Directly reported or could be inferred from the flow diagram.

## Discussion

### Principle findings of study

Our study provides a comprehensive assessment of the type and completeness of information reported in CONSORT flow diagrams published in current reports of randomized trials. Just over half (56%) of reports of randomized trials in our sample included a CONSORT flow diagram. This uptake might partly reflect the criteria for including journals in the National Library of Medicine core clinical journal list, and partly the large number of journals (79%) included in our sample which referred to the CONSORT Statement in their 'Instructions to Authors'. Indeed, one could argue that the number of reports of randomized trials including a flow diagram should be higher if those journals, which endorsed the CONSORT Statement, made inclusion of a flow diagram a requirement to journal submission. However, as has been shown previously, the language used in such endorsements varies markedly from requirement to mild encouragement [10].

The majority of reports of randomized trials which included a flow diagram were two-arm, parallel group trials investigating drugs as the primary intervention of interest. The average sample size was larger than that identified in a previous study conducted by our team which examined the reporting characteristics of randomized trials indexed in PubMed in 2006 [11], there the median sample size for parallel group trials was 80 versus 213 in our current study. That difference may well be a reflection of the journals tending to be more prestigious and higher impact than the full PubMed sample in the previous study.

A few elements of the flow diagram were well reported across trials; most published flow diagrams included the overall number of people assessed for eligibility, the overall number randomized, and the number allocated to the intervention per arm. However, only 40% of flow diagrams reported the number of participants (per arm) who actually received the intervention, and only 54% reported the number included in the main analysis. Details of the number of participants lost to follow up or discontinuing the intervention were also less well reported and authors sometimes combined these two items and did not differentiate between them. Some additional information was reported in the text, most often the reasons for exclusion and the numbers of participants included and excluded from the analysis, but this additional information did not greatly alter our findings and locating it required additional time and effort for the reader.

### Comparison with other studies

Several studies have looked at how often reports of randomized trials include a participant flow diagram and the type of information reported [4,12-16]. However, these studies are now out of date and have generally been restricted to trials in specific disease areas, journal types, or looked at specific elements of the flow of participants through the trial. A study of 207 randomized trials published in five general and internal medical journals in 1998 found that 51% of reports included a flow diagram; but this varied widely across journals [4]. Another study of randomized trials published in 1999 found that flow diagrams were more frequent in the Lancet (95%) and JAMA (86%) compared to the New England Journal of Medicine (13%) and BMJ (28%) [14].

One study looked specifically at the reporting of the recruitment process in 172 reports of randomized trials published in four high impact medical journals in 1999 to 2000. This found that 52% of flow diagrams reported the number of participants assessed for eligibility and only 43% reported the number eligible for participation in the trial [12]. In a more recent study of 113 reports of randomized trials published in six major journals in 2004, 79% of reports included a flow diagram, but over a third were incomplete. While the majority of flow diagrams reported the flow of participants at each stage of the trial after randomization, only 60% reported the number of participants assessed for eligibility [15]. Another study looking specifically at reports of randomized trials (n = 58), in the field of nutrition in pregnancy, found that while the majority (97%) of studies reported the number of participants allocated to each arm of the trial, only a third (31%) reported the number of participants assessed for eligibility [13].

These studies all show variability in the completeness and type of information reported and variation in their findings may primarily reflect changes over time and where the reports were published, with the majority of studies assessing a select group of high impact journals. Perhaps what is most interesting, when comparing the results of previous studies with those from our study, is the difference in reporting of the number of participants assessed for eligibility. Poor reporting of participant eligibility makes it difficult to know whether the enrolment process was highly selective and whether those who enrolled were representative of the general population [16,17]. Information on the overall number of participants assessed for eligibility has been poorly reported in previous studies but was reported in around 81% of flow diagrams included in our study. This could be an indication that trialists are now more likely to collect this level of detailed information as more journals ask authors to comply with the CONSORT Statement [10].

### Limitations of study

Our study has some limitations. We assessed only reports of trials which included a flow diagram. What we don't know is whether, in those trial reports which did not include a flow diagram, this type of information is available in the text and tables nor how long it would take a reader to locate this information; this will be subject to a future study. We limited our search to the National Library of Medicine's set of core clinical journals as these journals are typical of those accessed by most busy clinicians. This sample included a high proportion of journals which endorsed the CONSORT Statement which therefore should have included a CONSORT flow diagram. Therefore our findings might not be representative of all published reports of trials. We also recognise that there was a time lapse between publication of the trial reports in 2009 and when the journal 'Instruction to Authors' were assessed (November 2010). It is possible that a journal's endorsement of the CONSORT Statement may have changed during this time period; however, this is likely to be the case for very few trial reports and as such unlikely to significantly affect our conclusions

### Implications for practice

It is clear that although a substantial number of published trials now include a CONSORT flow diagram these are not always completed adequately. Endorsement of the CONSORT Statement by a journal is not sufficient, nor is the requirement that authors submit a flow diagram as part of their manuscript submission if it is not completed correctly. So what can be done to assist authors and journal editors? The recently revised CONSORT explanation and elaboration document is intended to enhance the use, understanding and dissemination of the CONSORT Statement; however it provides limited guidance in relation to the flow diagram [2], with the structure of the flow diagram last being revised in 2001 [4]. Improved clarification in the structure of the flow diagram, for example differentiating more clearly between loss to follow up and discontinuation of the intervention, would make it more logical for authors to complete and understand the importance of each item. For more complex trial designs one should also consider the benefits of including additional information in the flow diagram, such as the screening of participants prior to eligibility assessment, details of numbers followed up over multiple time points, or numbers included in different analyses, all of which were included in some flow diagrams in our study. In addition, development of a web based CONSORT flow diagram generator which automates the process of completing a flow diagram would help authors to complete all sections of the flow diagram appropriately (for example, https://depts.washington.edu/hrtk/CSD). This web based tool could be expanded to include both parallel group and multi-arms trials and also to take into account different study designs such as cluster or cross-over trials.

## Conclusions

Clear and detailed reporting of the flow of participants through each stage of the trial is essential in order to assess the validity and generalisability of the trial findings. Our study shows that, despite the majority of the trials included in our sample being published in a CONSORT endorsing journal, around 40% of reports of randomized trial did not include a flow diagram. Published flow diagrams often omitted some key aspects of the flow of participants through the trial, especially in relation to reasons for exclusion prior to randomization and, post randomization, reporting of the number of participants who actually received the allocated intervention, were lost to follow up or discontinued the intervention and those included in the main analysis. About half of these missing items were not available elsewhere in the article. If important information is not reported it can be difficult and sometimes impossible to know if the conclusions of that trial are justified by the data presented.

## Competing interests

SH and DGA are both members of the CONSORT Group. This study was carried out as part of a larger study funded by a grant from the UK National Institute for Health Research to support the work of the CONSORT Group. The funder had no role in the design, analysis, or interpretation of the study, or in writing of the manuscript.

## Authors' contributions

SH was involved in the design, implementation and analysis of the study and in writing of the final manuscript. AH, GC, SM, LY and DGA were involved in the design, implementation and analysis of the study and in commenting on drafts of the final manuscript. All authors read and approved the final manuscript.
